# Gene therapy following subretinal AAV5 vector delivery is not affected by a previous intravitreal AAV5 vector administration in the partner eye

**Published:** 2009-02-06

**Authors:** Wensheng Li, Fansheng Kong, Xia Li, Xufeng Dai, Xiaoqiang Liu, Qinxiang Zheng, Ronghan Wu, Xiangtian Zhou, Fan Lü, Bo Chang, Qiuhong Li, William W. Hauswirth, Jia Qu, Ji-jing Pang

**Affiliations:** 1 Eye Hospital, School of Optometry and Ophthalmology, Wenzhou Medical College, Wenzhou, China; 2 The Jackson Laboratory, Bar Harbor, ME; 3 Department of Ophthalmology, University of Florida, Gainesville, FL

## Abstract

**Purpose:**

In an earlier study we found normal adeno-associated viral vector type 2 (AAV2)-mediated GFP expression after intravitreal injection to one eye of normal C57BL/6J mice. However, GFP expression was very poor in the partner eye of the same mouse if this eye received an intravitreal injection of the same vector one month after the initial intravitreal injection. We also found both injections worked well if they were subretinal. In this study, we tested whether the efficiency of subretinal AAV vector transduction is altered by a previous intravitreal injection in the partner eye and more importantly whether therapeutic efficiency is altered in the *rd12* mouse (with a recessive *RPE65* mutation) after the same injection series.

**Methods:**

One μl of scAAV5-smCBA-GFP (1x10^13^ genome containing viral particles per ml) was intravitreally injected into the right eyes of four-week-old C57BL/6J mice and 1 μl of scAAV5-smCBA-hRPE65 (1x10^13^ genome containing viral particles per ml) was intravitreally injected into the right eyes of four-week-old *rd12* mice Four weeks later, the same vectors were subretinally injected into the left eyes of the same C57BL/6J and *rd12* mice. Left eyes of another cohort of eight-week-old *rd12* mice received a single subretinal injection of the same scAAV5-smCBA-hRPE65 vector as the positive control. Dark-adapted electroretinograms (ERGs) were recorded five months after the subretinal injections. AAV-mediated GFP expression in C57BL/6J mice and RPE65 expression and ERG restoration in *rd12* mice were evaluated five months after the second subretinal injection. Frozen section analysis was performed for GFP fluorescence in C57BL/6J mice and immunostaining for RPE65 in *rd12* eyes.

**Results:**

In *rd12* mice, dark-adapted ERGs were minimal following the first intravitreal injection of scAAV5-smCBA-RPE65. Following subsequent subretinal injection in the partner eye, dramatic ERG restoration was recorded in that eye. In fact, ERG b-wave amplitudes were statistically similar to those from the eyes that received the initial subretinal injection at a similar age. In C57BL/6J mice, GFP positive cells were detected in eyes following the first intravitreal injection around the injection site. Strong GFP expression in both the retinal pigment epithelium (RPE) and photoreceptor (PR) cells was detected in the partner eyes following the subsequent subretinal injection. Immunostaining of retinal sections with anti-RPE65 antibody showed strong RPE65 expression mainly in the RPE cells of subretinally injected eyes but not in the intravitreally injected eyes except minimally around the injection site.

**Conclusions:**

These results show that an initial intravitreal injection of AAV vectors to one eye of a mouse does not influence AAV-mediated gene expression or related therapeutic effects in the other eye when vectors are administered to the subretinal space. This suggests that the subretinal space possesses a unique immune privilege relative to the vitreous cavity.

## Introduction

Immune privilege is one of the important features of the eye, which makes it an attractive target for gene therapy. The posterior part of the eye also appears to have immune deviant features. Streilein et al. [1–4] reported an immune-deviant response against soluble and cell-bound antigens in the subretinal space. The anatomic structure of the eye may assist in mediating immune deviation including the fact that much of the eye is avascular. In addition, there are several cellular and physical barriers, which enforce the separation from the blood supply.

Adeno-associated virus (AAV) is a human parvovirus, which has not been associated with human disease [5]. It has favorable immunologic characteristics as a vector after deleting all viral open reading frames and retaining only the inverted terminal repeat sequences (ITRs) [6]. Exposure to recombinant AAV has not been reported to induce a cell-mediated immune response in the eye. However, this virus can induce a strong antibody response directed at both viral capsid antigens and the transgene [7–9]. Antibodies are detected in the intraocular fluid (anterior chamber fluid and vitreous) as well as in the serum.

Although both the vitreous cavity (VC) and subretinal (SR) spaces possess immune privilege, the VC space behaves differently to AAV-mediated gene transfer than the SR space [10]. The VC space is capable of eliciting an immune response against AAV capsid while the SR space is not. The precise mechanism remains unknown. However, it is possible that the VC outflow mechanisms and its close proximity to vascular systems play critical roles in the immune response. The SR space is a potential space between the retinal pigment epithelium (RPE) and photoreceptor (PR) cells. The RPE monolayer forms the outer blood-retina barrier (BRB), separating the choroicapillaris from the neural retina, and controls the exchange of molecules between the retina and choroid [10]. RPE cells can also secrete different immune-suppressive and anti-inflammatory molecules as well as cell membrane-bound molecules, which can induce apoptosis of inflammatory cells and contribute to ocular immune privilege [11–14].

Studies of repeated administration of AAV vectors into non-ocular tissue indicate that immune responses generated after the first administration may prevent further application [15–20]. While it is generally assumed that pre-exposure to AAV will not pose significant problems for the efficacy of AAV vectors in the retina, an immune privileged site, no study had been carried to examine the impact of previous intravitreal injection in one eye on subsequent AAV vector-mediated therapy in the second eye. Recently, we found that an intravitreal injection of AAV2-CBA-pigment epithelium-derived factor (PEDF) into C57-BL/6J mice resulted in a humoral immune response against AAV2 capsid, which prevented therapy when the same vector was re-administrated to the VC of the contralateral eye [10]. In a parallel study, we found normal AAV2-mediated GFP expression after an intravitreal injection to one eye of normal C57BL/6J mice. In contrast, GFP expression was very poor in the other eye of the same mouse if it received a subsequent intravitreal injection of the same vector one month after the initial intravitreal injection in the partner eye. However, both injections worked well if they were subretinal [10].

Since the Leber congenital amaurosis (LCA2, with *RPE65* mutation) clinical trial began and only one eye was treated each time [21–24], this raises the concern that an initial subretinal injection may affect the future therapeutic effect of later treatments in the contralateral eye of that patient. This concern stems from the possible chance that some AAV vector might leak into the vitreous cavity during or after the original subretinal injection procedure.

Recently, a naturally occurring mouse model of human LCA with *RPE65* mutations, the *rd12* mouse, was reported [25]. In this model, a recessive nonsense mutation in *Rpe65* leads to the absence of the RPE65 protein and blockage of the retinoid cycle, which is essential for rod function. Because of undetectable levels of RPE65, 11-cis-retinal, and rhodopsin at any age, no normal, dark-adapted electroretinogram (ERG) was detected in *rd12* mice and slow rod degeneration ensued [25]. RPE65, 11-cis retinal, and rod ERGs were restored following subretinal delivery of either AAV2-RPE65 or AAV5-RPE65 at different ages of RPE65 deficient mice [26–29].

In this study, we used the *rd12* mouse to test whether the efficiency of subretinal AAV-RPE65 therapy is altered by a previous intravitreal treatment of the partner eye with the same vector and whether we can restore ERG function in the subretinally treated *rd12* eye.

## Methods

### Animals

C57BL/6J mice and the congenic inbred strain of *rd12* mice were obtained from the Jackson Laboratory (Bar Harbor, ME) and bred at Wenzhou Medical College (Wenzhou, China). All mice were maintained in the Animal Facilities of Wenzhou Medical College under a 12-h light/12-h dark cycle. Sixty mice were used in this study. All experiments were approved by the Wenzhou Medical College’s Institutional Animal Care and Use Committee and were conducted in accordance with the ARVO Statement for the Use of Animals in Ophthalmic and Vision Research.

### Adeno-associated virus type 5 vector preparation

The vector plasmid of self-complementary (sc) human RPE65 (hRPE65) with a small, hybrid cytomegalovirus (CMV)-chicken β-actin (smCBA) promoter (sc-smCBA-hRPE65) was constructed by replacing the humanized green fluorescent protein (GFP) cDNA of sc-smCBA-hGFP with the human RPE65 cDNA via flanking Not I sites. The vector plasmid for scAAV5-smCBA-hRPE65 was constructed by replacing the humanized GFP cDNA of sc-smCBA-hGFP with the human RPE65 cDNA, via a Not I digest. Both constructs contain flanking AAV serotype 2 inverted terminal repeats (ITR); one ITR has modifications required for packaging as a self complementary AAV vector [30]. Pseudotyped AAV5 capsid, self-complementary AAV5 vectors (scAAV5) were used in this study as they have been shown to be more efficient vectors for transduction of the retina than standard, single-stranded AAV vectors [31,32]. The therapeutic vector (scAAV5-smCBA-hRPE65) has been shown to have identical transduction and tropism characteristics as the full chimeric CMV-CBA promoter when targeted to the mouse retina [33]. Vectors were manufactured by previously described methods [34]. Viral preparations had an average titer of 10^13^ genome-containing viral particles per ml. The vector titer was determined by real time polymerase chain reaction (PCR), and final aliquots were resuspended in balanced salt solution (Alcon Laboratories, Forth Worth TX) with 0.014% Tween-20 (J.T. Baker, Inc., Phillipsburg, NJ).

### Subretinal and intravitreal injections

One μl of scAAV5-smCBA-GFP (1x10^13^ genome containing viral particles per ml) was intravitreally injected into the right eyes of four-week-old C57BL/6J mice and 1 μl of scAAV5-smCBA-hRPE65 (1x10^13^ genome containing viral particles per ml) was intravitreally injected into the right eyes of four-week-old *rd12* mice. Four weeks later the same vectors were subretinally injected into the left eyes of the same C57BL/6J or *rd12* mice using a previously described method [26]. Ten eight-week-old *rd12* mice received a single subretinal injection to their right eyes with the same scAAV5-smCBA-hRPE65 vector as the positive control.

For subretinal injections, 1 μl vector suspension with 1% fluorescein, diluted from 25% AK-FLUR (Akorn, Buffalo Grove, IL) was slowly injected subretinally. The injected retinal area was visualized by fluorescein-positive subretinal blebs demarking the retinal detachment. Such detachments were usually resolved within 24 h. Signs of injection-related damage included large holes in the cornea with accompanying iris–cornea adhesion, hemorrhage in the iris or retina, and damage to the lens, causing cataract formation. Animals with any of these complications were removed from further study. In animals with no apparent surgical complications, only those whose retinal blebs occupying more than 90% of the retina were retained for further evaluation. Twenty mice met this standard in the study, which included three *rd12* mice that received only a single subretinal injection for ERG examination, three *rd12* mice that received an intravitreal injection followed by a subretinal injection in the partner eyes for ERG examination, six *rd12* mice that received an intravitreal injection followed by subretinal injection in partner eyes for immunostaining, and eight C57BL/6J mice that received intravitreal injection followed by subretinal injection in partner eyes for retinal whole-mount and sectioning examinations. One drop of 1% atropine (Hi-Tech Pharmacal Co. Inc., Amityville, NY) and a small amount of Neomycin and Polymyxin B Sulfates and Dexamethasone Ophthalmic Ointment (E. Fougera and Co., Melville, NY) were applied to the eye following injection and then applied once daily for three days to prevent the injection-related inflammation and iris-cornea adhesion as well as bacteria and fungi infections. Evaluation was performed five months after the second SR injection.

### Electroretinogram recording and statistical analysis

Nine *rd12* and nine C57BL/6J mice were dark adapted overnight and anesthetized with a mixture of ketamine (15 mg/g) and xylazine (5 mg/g bodyweight) under dim red light. The pupils were dilated with a single drop of 1% atropine sulfate. A drop of 0.5% proparacaine hydrochloride was applied for corneal anesthesia. The temperature of the room was maintained at 38 °C. A small amount of 2.5% methylcellulose gel was applied to the eye. A silver loop electrode was placed over the cornea to record the ERGs. Needle reference and ground electrodes were inserted into the cheek and tail, respectively. The responses were differentially amplified (1–500Hz). All stimuli were presented in a Ganzfeld dome (Roland Q400, Wiesbaden, Germany). Light was spectrally filtered with a 500-nm interference filter. Flashes varied in intensity from −5.0 to 0 log scotopic candela-sec/m^2^. For reporting b-wave amplitudes, data in each group (n=3) was expressed as mean ±standard deviation (SD), and the significant difference was judged by one-way Analysis of Variance (ANOVA). Least Significant Difference (LSD) was then used for a post hoc test.

### Histology

Mouse eyes were enucleated and fixed overnight with 4% paraformaldehyde in phosphate-buffered saline (dPBS; Mediatech, Inc., Herndon, VA) after removal of the cornea. Eyecups were then prepared by removing the lens. Retinal whole-mounts were prepared by removing the choroid and sclera from the eyecups. The whole-mounts were then placed on a slide (photoreceptors down) and coverslipped before photographing. Frozen sections were prepared from eyecups, rinsed four times in dPBS, transferred to 30% sucrose in dPBS for 5 h, and frozen in optimal-cutting temperature medium (Fisher Scientific, Pittsburgh, PA). Twelve-micrometer thick sections were obtained with a cryostat. GFP-specific fluorescence was analyzed using an Olympus CK40 inverted microscope (Olympus, Tokyo, Japan). True GFP fluorescence was distinguished from background autofluorescence by comparing signals through a fluorescein isothiocyanate (FITC) and a rhodamine filter. Sections expressing GFP were covered with Vectashield mounting medium (Vector Laboratories, Burlingame, CA) and photographed using a Spot RT (real-time) digital camera (Diagnostic Instruments Color Digital Cameras, McHenry, IL).

### Immunocytochemistry for RPE65

Eyes from C57BL/6J mice and from both injected eyes of *rd12* mice at seven months of age were used to prepare frozen sections as described above. Following permeabilization with 0.1% Triton X-100, 12-μm thick frozen sections mounted on coated slides were rinsed with dPBS (Mediatech, Inc., Herndon, VA), blocked with 20% normal goat serum (NGS), incubated overnight at 4 °C in rabbit polyclonal raised anti-human/bovine RPE65 antibodies (Abcam, Cambridge, UK), and diluted 1:400 in NGS. After three rinses with dPBS, sections were incubated in goat anti-rabbit Texas red (1:300, Molecular Probes, Eugene, OR) for 2 h followed by three rinses with dPBS. Sections were then mounted with coverslips for fluorescence photography.

## Results

### Effect of vector expression following intravitreal and subretinal injection of scAAV5-smCBA-GFP to C57BL/6J mice

**Figure 1:**
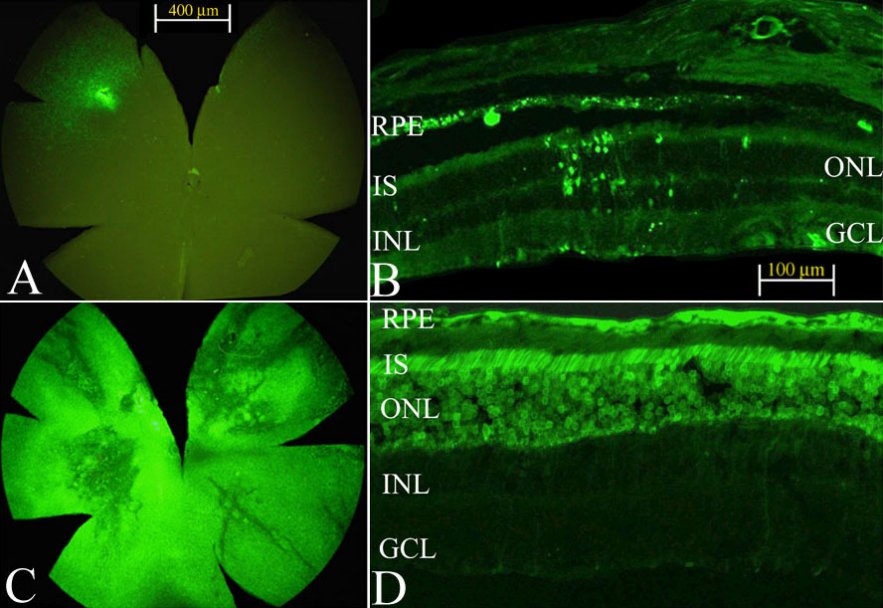
AAV5-mediated GFP expression both in retinal whole mounts and frozen sections following intravitreal and subretinal injection of scAAV5-smCBA-GFP. Higher transduction efficiency is seen following subretinal injection compared to intravitreal injection. **A**: Fluorescence image of a retinal whole-mount six months following intravitreal injection. **B**: Fluorescence image of a frozen section six months following intravitreal injection. **C**: Fluorescence image of a retinal whole-mount five months after subretinal injection (the partner eye of this mouse was pretreated with the same vector by intravitreal injection one month earlier). **D**: Fluorescence image of a frozen section as in (**C**). Abbreviations: RPE represents retinal pigment epithelium; IS represents inner segments of photoreceptor cell; ONL represents outer nuclear layer; INL represents inner nuclear layer; GCL represents ganglion cell layer.

Retinal whole-mount analysis showed minimal transduction in the retina mainly around the injection site six months following the first intravitreal injection of scAAV5-smCBA-GFP (Figure 1A). Fluorescent microscopy showed that those GFP positive cells were mainly RPE and PR cells (Figure 1B). Very few GFP positive retinal ganglion cells (RGCs) could be detected, and the few detected were found only around the injection site. In contrast, retinal whole-mounts subretinally injected with the same AAV5 vector showed strong GFP expression throughout the entire retina five months following the second subretinal injection (Figure 1C). Fluorescent microscopy of transverse retinal sections showed strong GFP expression mainly in the RPE and PR cells after subretinal injection of scAAV5-smCBA-GFP (Figure 1D). *Rd12* mice in which only one eye received subretinal scAAV5-smCBA-GFP at eight weeks of age showed similar results as those shown in Figure 1C,D (data not shown).

### RPE65 expression following intravitreal and subretinal injections of scAAV5-smCBA-hRPE65 vector into *rd12* mice

**Figure 2:**
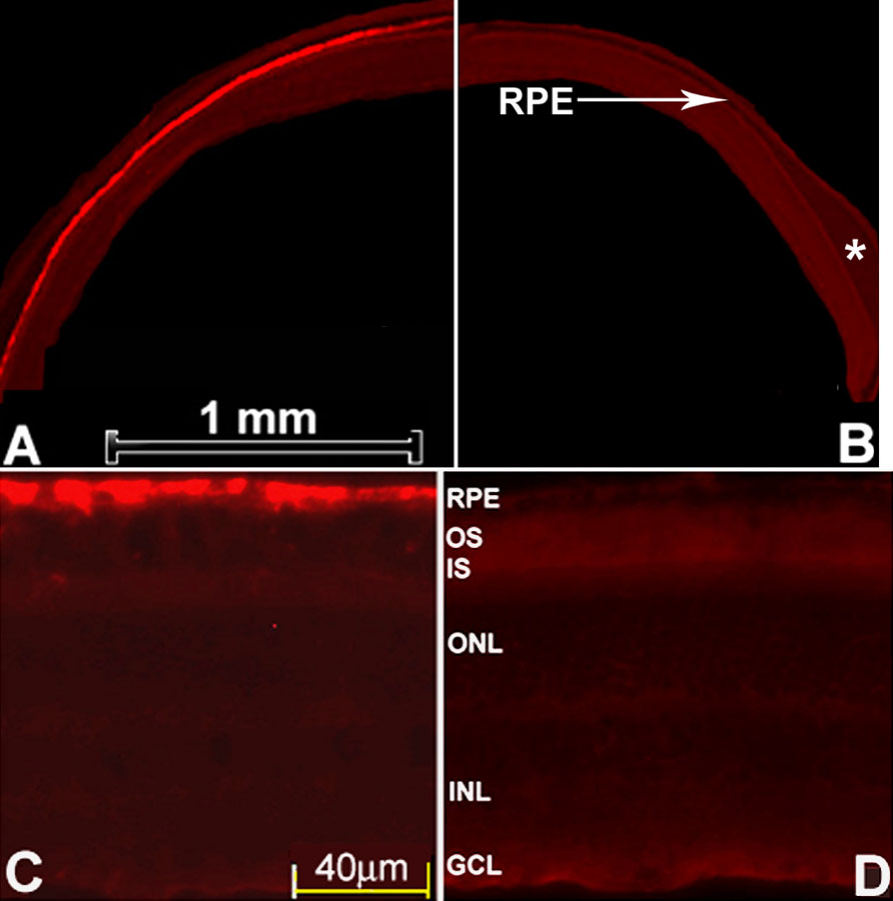
RPE65 immunoreactivity after intravitreal and subretinal injections of scAAV5-smCBA-hRPE65 vector. Little RPE65 expression was detected in an *rd12* eye six months after the intravitreal injection (**B**), which was further supported by a higher magnification image (**D**). RPE65 expression was detected in the RPE cells of the contralateral eye from the same *rd12* mouse five months after subretinal injection (**A**), which was further supported by a higher magnification image (**C**). Abbreviations: RPE represents retinal pigment epithelium; OS represents outer segments of photoreceptor cell; IS represents inner segments of photoreceptor cell; ONL represents outer nuclear layer; INL represents inner nuclear layer; GCL represents retinal ganglion cell layer. The asterisk shows retinal detachment.

Stable RPE65 expression throughout the whole retina was observed in the *rd12* eyes five months after the subretinal injection of scAAV5-smCBA-hRPE65 (Figure 2A). In the contralateral eyes, which received an intravitreal injection with the same vector four weeks before the subretinal injection, little RPE65 expression could be detected (Figure 2B) except modest expression around the injection site (data not shown). High magnification fluorescence microscopy (Figure 2C) showed that RPE65 was mainly located in the RPE cells of the *rd12* eye following the subretinal injection of scAAV5-smCBA-hRPE65. In the partner *rd12* eye that received an intravitreal injection, no obvious RPE65 expression was detected (Figure 2D). Occasionally, a weak signal could also be detected in the RPE and PR cells under high magnification around the injection site (data not shown).

### Electroretinographic analysis

**Figure 3:**
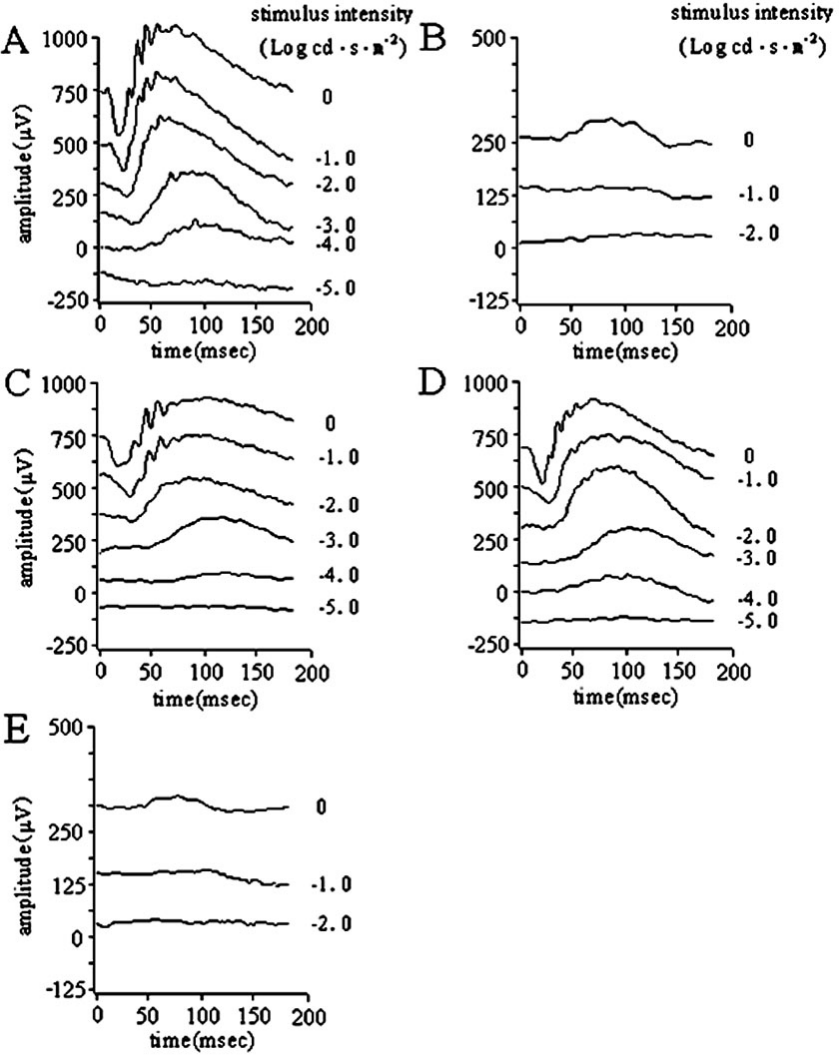
Dark-adapted (rod-derived) ERG analysis showing retinal function of seven-month-old normal and *rd12* mice following either intravitreal or subretinal injection, or no injection. Restored ERGs are independent of whether the partner eye was pretreated with the same vector or not. Dark-adapted photoresponses are shown at different input flash intensities from an uninjected normal C57BL/6 (**A**) and an uninjected *rd12* eye (**B**). Panel **C** shows the ERGs from the left eye of an *rd12* mouse five months after subretinal injection at eight weeks of age, while the right eye of the same mouse had received intravitreal vector four weeks before the subretinal injection (**E**). Panel **D** shows ERGs from an *rd12* eye five months following only a subretinal injection at eight weeks of age with no prior treatment of the partner eye.

**Figure 4:**
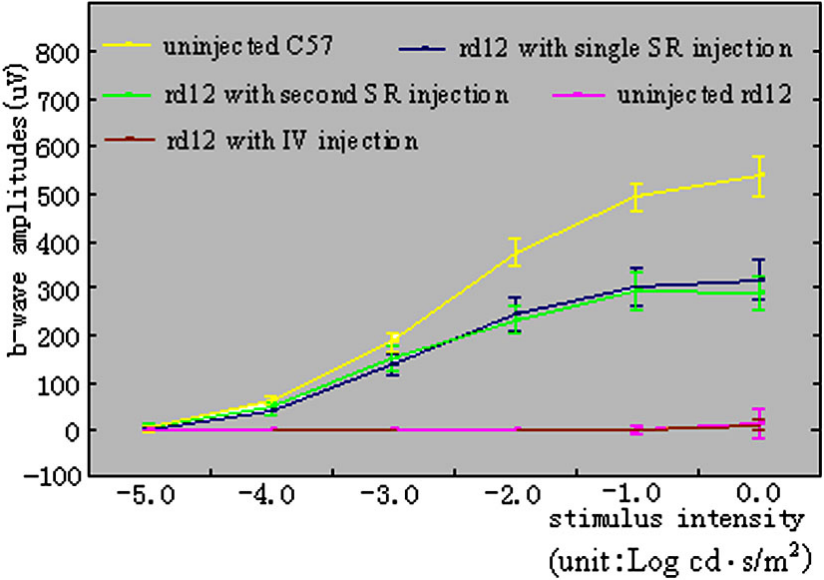
ERG signals following different injections. Dark-adapted ERG b-wave amplitudes under different intensities show dramatic ERG restoration in rd12 mice 5 months following subretinal injection. Statistical analysis demonstrates that functional restoration following subretinal injection is independent of whether the partner eye was pretreated with the same vector or not. Each of the five groups had three mice. The yellow curve represents the uninjected, normal C57BL/6J eyes at seven months of age. The blue curve represents the *rd12* eyes at seven months of age following an initial subretinal injection of scAAV5-smCBA-hRPE65 when they were eight weeks old. The brown curve represents the right eyes of *rd12* mice six months following the intravitreal injection that occurred when they were four weeks old. The green curve represents the left eyes of *rd12* mice five months following the subretinal injection they received when they were eight weeks old (the right eyes of these mice received the intravitreal vector four weeks before their subretinal injections). The pink curve represents the untreated *rd12* eyes at seven months of age. Bars: mean±SD.

A series of ERG responses from seven-month-old, uninjected, normal C57BL/6J, and *rd12* eyes are shown in Figure 3A,B, respectively. The *rd12* eye, which received a subretinal injection four weeks after an intravitreal injection in the contralateral eye of the same mouse (Figure 3C), showed robust, dark-adapted ERG restoration five months after the subretinal injection. The amplitude was similar to the *rd12* eye that received only one subretinal injection at a similar age (Figure 3D). In contrast, the intravitreally injected *rd12* eye (Figure 3E, contralateral eye of Figure 3C) showed little or no dark-adapted ERG signal, similar to the age-matched uninjected *rd12* eye (Figure 3B). The b-wave amplitudes (Figure 4) from each experimental group are 536.7±41.1 μV (maximum response) in normal, seven-month-old C57BL/6J eyes, 293.7±26.8 μV (maximum response) in *rd12* eyes five months following a subretinal injection at eight weeks old (four weeks following intravitreal injection in the partner eye), 317.3±42.2 μV (maximum response) in *rd12* eyes five months following a single subretinal injection at eight weeks old, 16.7±28.9 μV (maximum response) in seven-month-old, untreated *rd12* eyes, and 15.0±9.0 μV (maximum response) in *rd12* eyes six months following intravitreal injection at four weeks old. The maximum b-wave amplitudes (Figure 4) in subretinally treated *rd12* eyes were about 55% of those in the normal, uninjected C57BL/6J eyes. Statistical analysis (Figure 4) showed that *rd12* eyes that received either a second subretinal injection (n=3, p<0.001) or a single subretinal injection (n=3, p=0.001) had significantly higher b-wave amplitudes than the untreated *rd12* eyes. However, treated *rd12* eyes had lower b-wave amplitudes than those in the normal, uninjected C57BL/6J eyes (n=3, p<0.001). Although the average amplitude of b-waves in eyes receiving the second subretinal injection were slightly lower than in eyes receiving a single subretinal injection, there was no statistically significant difference between these two groups (n=3, p=0.458). Also, there was no statistical difference in b-wave amplitudes from seven-month-old *rd12* eyes either receiving intravitreal injection at four weeks old (n=3) or receiving no injection (n=3, p=0.944).

## Discussion

AAV vectors have many attractive features for safe and efficient gene therapy including their lack of pathogenesis, low toxicity, ability to efficiently infect both dividing and non-dividing cells in broad range of host tissues/organs, and long-term gene expression [35,36]. The ability of AAV vectors to efficiently transduce retinal cells has been exploited to successfully transfer therapeutic genes into PR, RPE, and RGCs [37], to treat a variety of retinal diseases causing blindness in animal models [26,38,39] and now in human LCA [21–24].

A key consideration in the design of the delivery vector for gene therapy is the AAV serotype. Adeno-associated virus type 5 (AAV5) is one of the most divergent of the AAV serotypes, sharing only 64% overall nucleotide identity with the prototype AAV2 [40,41]. While the basic transcription profile of AAV5 is similar to that of AAV2, there are also significant differences [42]. In contrast to AAV2 that can efficiently transduce the ganglion cells following intravitreal injection, AAV5 vectors only transduce retinal cells inefficiently following intravitreal injection [37]. It is difficult to compare ERG restoration differences in the two *rd12* groups even when both received subretinal injections because the retinal transduction areas vary unpredictably with each subretinal injection. Therefore, we only used mice that had no injection related complications and had at least 90% of the retina detached upon subretinal injection.

In parallel experiments, we found that *rd12* eyes showed similar rod-related ERG restoration following either an initial subretinal injection of scAAV2-smCBA-RPE65 or a second subretinal injection when the other eye had received an intravitreal injection of the same vector four weeks before. However, we also noted that a subretinal injection of scAAV5-smCBA-RPE65 led to better ERG restoration than scAAV2-smCBA-RPE65 and that scAAV5-smCBA-hRPE65 was slightly better than the corresponding vector containing a 0.9 kb larger CBA promoter, AAV5-CBA-hRPE65 (data not shown). Thus, serotype 5 scAAV-smCBA-RPE65 was chosen as the delivery vector over serotype 2 because it can transfect more RPE and PR cells [26,37,43,44] and yield more RPE65 protein at the desired site.

Immunohistochemical data reported here suggest that the AAV5-mediated delivery of human RPE65 results in sustained human RPE65 protein expression in the *rd12* retina for at least five months that correlates with a sustained restoration of dark-adapted ERG signals. RPE65 expression and ERG restoration has been detected as early as four weeks following subretinal injection (data not shown) and remained stable for at least five months after subretinal injection, the longest time we observed in this study. This biochemical and functional restoration was independent of whether the partner eye was pretreated or not with the same vector and suggests that AAV-mediated RPE65 expression and therapy following subretinal injection is not modified by previous intravitreal injection in the partner eye.

It is known that following intravitreal injection of AAV vector, a systemic antibody will form that can then alter the transduction efficacy of inner retinal cells in the partner eye upon a subsequent intravitreal vector injection [10]. In that study, a strong humoral immune response against AAV capsid was observed four weeks after intravitreal treatment with AAV5-RPE65 vector. This was the main reason for choosing this specific timing between contralateral intravitreal and subretinal injections. Therefore, our principal finding was therapeutic. AAV-mediated RPE65 expression and ERG functional rescue were not reduced if the subretinal injection was preceded by an intravitreal injection of the same vector into the contralateral eye. Indeed, there is no reduction of the therapeutic effect (as determined by ERG amplitudes in the subretinally injected eye) no matter whether the partner eye was pretreated with the same vector or not. This suggests that, as far as RPE targeted therapy is concerned, the subretinal space possesses full immune privilege.

Our results are consistent with those of Bennett et al. [45] and Anand et al. [46] in which non-human primate and murine RPE and PR cells were shown to be transduced efficiently by subretinal injection of AAV vectors in spite of the presence of circulating antibodies to AAV capsid. Furthermore, our findings have added clinical implications for the design of gene therapy protocols aimed either at targeting different retinal cell types in different ocular compartments of the same eye or at treating partner eyes sequentially. Although care must be taken in extrapolating results in mice to humans, particularly under pathological conditions, our results suggest that it is safe to re-administer AAV vectors into the subretinal space to target PR and RPE cells without compromising the efficacy of the repeated gene transfers.
